# Dual PI3K/mTOR Inhibitor BEZ235 combined with BMS-1166 Promoting Apoptosis in Colorectal Cancer

**DOI:** 10.7150/ijms.84320

**Published:** 2024-07-09

**Authors:** Xueke Liu, Wei Xu, Lele Li, Zhenyong Zhang, Mei Lu, Xiaoping Xia

**Affiliations:** Department of Clinical Laboratory, the Fourth Affiliated Hospital of School of Medicine, and International School of Medicine, International Institutes of Medicine, Zhejiang University, Yiwu, China, 322000.

**Keywords:** BEZ235, BMS-1166, PD1/PD-L1, signaling pathway, apoptosis, colorectal cancer

## Abstract

**Background:** BMS-1166, a PD-1/PD-L1 inhibitor, inhibits the binding of PD-L1 to PD-1, restores T cell function, and enhances tumor immune response. However, mutations in the tumor suppressor or impaired cellular signaling pathways may also lead to cellular transformation. In this study, the SW480 and SW480R cell lines were used as the model to elucidate the treatment with BMS-1166, BEZ235, and their combination.

**Methods:** MTT and colony-formation assays were used to evaluate cell proliferation. Wound-healing assay was used to assess cell migration. Cell cycle and apoptosis were analyzed by flow cytometry. The phosphorylation level of the key kinases in the PI3K/Akt/mTOR and MAPK pathways, PD-L1, and the protein levels related to the proliferation, migration, and apoptosis were assessed using western blotting.

**Results:** BEZ235 enhanced BMS-1166-mediated cell proliferation and migration inhibition in SW480 and SW480R cells and promoted apoptosis. Interestingly, the downregulation of the negative regulator PTEN raised the PD-L1 level, which was abolished by the inhibition of Akt. BMS-1166 promoted PI3K, Akt, mTOR, and Erk phosphorylation. However, the combination of BEZ235 with BMS-1166 suppressed the expression of PI3K, p-Akt, p-mTOR, and p-Erk in SW480 and SW480R cells compared to BMS-1166 or BEZ235 single treatment by inhibiting the binding of PD1 to PD-L1.

**Conclusions:** PD-1 binds to PD-L1 and activates the PI3K/mTOR and MAPK pathways, which might be the molecular mechanism of acquired resistance of CRC to BMS-1166. The combination of the two drugs inhibited the phosphorylation of PI3K, Akt, and Erk in the PI3K/mTOR and MAPK pathway, i.e., BEZ235 enhanced the BMS-1166 treatment effect by blocking the PI3K/mTOR pathway and interfering with the crosstalk of the MAPK pathway. Therefore, these findings provide a theoretical basis for BMS-1166 combined with BEZ235 in the trial treatment of colorectal cancer.

## Introduction

Colorectal cancer (CRC) is the third leading cause of cancer-related deaths after lung and breast cancers, and thus a major public health issue worldwide^1^. Currently, the treatments for CRC include surgery, chemotherapy, radiotherapy, and targeted therapy^2^. Previous studies indicated that apoptosis resistance is one of the main reasons for resistance during tumor therapy^3^,^4^. As the incidence of CRC increases each year, immunotherapy plays a vital role in the overall survival of the patients. In recent years, radiotherapy and antibody therapies have prolonged the onset of disease recurrence and delayed metastasis. However, the development of CRC is a complex multifactorial, multistep, multistage progression, due to which the mortality rate remains high^5^. Therefore, the potential molecular mechanisms of chemotherapy resistance in the progress of CRC need to be elucidated to develop effective targeted therapy, reduce the recurrence rate, and eliminate drug resistance.

**Figure 1:**
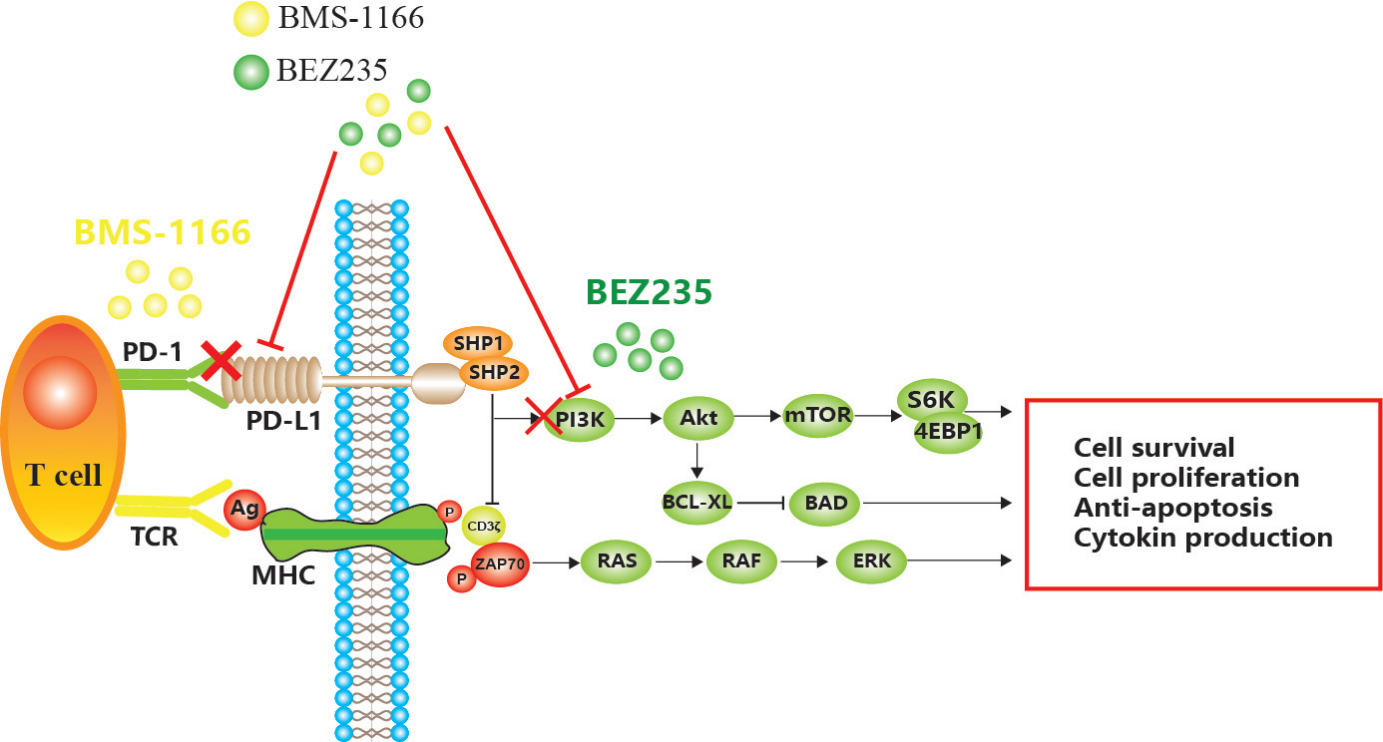
Mechanism of apoptosis induced by BEZ235 and BMS-1166 in CRC cells.

BMS-1166 is an inhibitor of PD-1/PD-L1 interaction. It has low toxicity to the tested cell lines, inhibits the interaction between programmed cell death ligand (PD-L1) and programmed cell death receptor (PD-1), reduces the inhibitory effect of PD-L1 on T cell receptor-mediated T lymphocyte activation, and enhances tumor immune response, thus exerting anticancer effect^6^,^7^,^8^. However, with prolonged treatment, CRC becomes resistant to BMS-1166, leading to the phosphorylation of tyrosine residues in the cytoplasmic region of immune receptor tyrosine conversion motif (ITSM) domain induced by PD-L1 binding to PD-1 and the recruitment of protein tyrosine phosphatase 2 (SHP-2) in the Src homologous domain. SHP-2 feedback activates the downstream PI3K/mTOR and MAPK pathway^9^,^10^,^11^. Some miRNAs indirectly promote the expression of PD-L1 by inhibiting PTEN, while decreased PTEN levels lead to the abnormal activation of the PI3K/mTOR pathway, and activated mTOR stabilizes the protein level of PD-L1^12^,^13^. Akt regulates cell proliferation and survival. PDK1-stimulated Akt phosphorylates many protein targets on the membrane, such as caspase-9 and mTOR^14^. mTOR is a serine/threonine kinase that binds to other proteins to form the mTOR complex 1 (mTORC1) or mTOR complex 2 (mTORC2)^15^. mTOR induces mRNA translation by phosphorylating S6K, accelerates the cell cycle, promotes tumor cell migration, suppresses apoptosis, and leads to CRC tolerance and tumor immune escape^16^ (**Figure 1**).

BEZ235 is an orally bioavailable imidazoquinoline derivative that inhibits the activity of PI3K/mTOR. The therapeutic potency of BEZ235 is efficacious as a monotherapeutic drug or in combination with other anticancer drugs^17^. Specifically, BEZ235 treatment enhances the sensitivity to other drugs and improves drug resistance in several malignancies, including acute myeloid ovarian cancer, non-small cell lung cancer, and gastric cancer^18^.

In summary, we speculated that by inhibiting the combination of PD1 and PD-L1, the function of T cells can be restored, which inhibits the abnormal activation of PI3K/mTOR and MAPK pathways, reduces CRC cell proliferation/migration, and promotes apoptosis. These findings might provide a novel idea and technical platform for the elimination of drug resistance.

## Materials and methods

### Reagents and antibodies

BMS-1166 and PI3K/mTOR inhibitor (BEZ235) were obtained from MedChem Express (Monmouth Junction, NJ, USA). 5,5',6,6'-tetrachloro-1,1',3,3'-tetraethyl-benzimidazolyl carbocyanine iodide (JC-1), 3-(4,5-dimethyl-2-thiazolyl)-2,5-diphenyl-2-H-tetrazolium bromide (MTT), and Annexin V-FITC/propidium iodide (PI) Kit were purchased from Sigma-Aldrich (St. Louis, MO, USA). All antibodies were obtained from Cell Signaling Technology (Danvers, MA, USA).

### Cell lines and cell culture

SW480 cell line was purchased from American Type Culture Collection (Rockville, MD, USA). SW480R, an acquired BMS-1166-resistant CRC cell line, was established from SW480 cells as follows: The culture medium was changed when the cells were in the logarithmic growth phase, and a lower concentration of BMS-1166 was added for 24 h. During cell passage, and stimulation with difference concentration was repeated until it was stable. The resistant strain was obtained when the BMS-1166 concentration reached 4-5 times the half-maximal inhibitory concentration value of the sensitive strain. The two cell lines were cultured in RPMI-1640 (Hyclone, Salt Lake City, UT, USA), supplemented with 10% fetal bovine serum (FBS; Sijiqing Bioengineering Materials, Hangzhou, China), at 37 °C under 5% CO_2_.

### MTT assay

Cells were seeded in 96-well plates at 4×10^4^ cells/mL in 100 μL of RPMI-1640 plus 10% FBS and cultured for 48 h at 37 °C in a 5% CO_2_ incubator. Then, the cells were treated with various concentrations of agents for 24 h. Subsequently, 10 μL of MTT (5 mg/mL) was added to each well and incubated for 4 h. The reaction was quenched with 100 μL dimethyl sulfoxide. The optical density was measured at 490 nm on a microplate reader. The inhibitory effects of BEZ235 combined with BMS-1166 on SW480 and SW480R cells were calculated.

### Colony formation assay

Cells were plated in six-well plates at a density of 2×10^3^/well for 24 h. After treatment with various concentrations of drugs for another 24 h, the drug-containing medium was replaced with drug-free medium, and the cells were cultured for an additional two weeks. When the colonies were visible, the cells were fixed in 4% paraformaldehyde for 15 min and stained with 0.1% crystal violet for 30 min for counting. The rate of colony formation reflects the cell proliferation ability. All experiments were carried out in triplicate.

### Wound healing assay

Cells (2×10^5^/well) were plated in six-well plates in RPMI-1640 containing 10% FBS until confluency. The cell monolayer was scratched with a sterile 10-μL tip to create a cell-free zone. After the cells were washed with phosphate-buffered saline (PBS) three times, serum-free medium containing drugs was added to corresponding wells and incubated at 37 °C in a humidified atmosphere under 5% CO_2_. The width of the scratched area was recorded at 0, 12, 24, and 48 h by images captured under a light microscope.

### Mitochondrial membrane potential (ΔΨm) measurement

JC-1 is a cationic dye that accumulates in the mitochondria, indicated by a fluorescence emission shift from red (~590 nm) to green (~525 nm), and can be used as a dual-emission potential-sensitive probe for measuring ΔΨm. SW480 and SW480R cells were cultured in 24-well plates overnight, treated with drugs for 24 h, counterstained with DAPI and JC-1, and examined under a fluorescence microscope. SW480 and SW480R cells treated with the drug were stained with 10 μg/mL JC-1 at room temperature for 30 min and analyzed for changes in ΔΨm by flow cytometry (BD Biosciences).

### Apoptosis detection

To detect apoptosis, cells were seeded on pretreated glass slides in 24-well plates at a density of 1×10^3^ cells/well and incubated overnight, followed by drug treatment for an additional 24 h. After washing with PBS, the cells were incubated with 1 μL fluorescence DNA stain (DAPI), 5 μL AnnexinV-FITC, and 5 μL PI at room temperature for 15 min in the dark. The apoptosis rate was analyzed under a fluorescence microscope.

An equivalent of 1×10^5^ cells/well was seeded overnight on slides in six-well plates. After drug treatment for 24 h, the cells from the experimental groups were collected, washed with cold PBS, resuspended in 500 μL binding buffer containing 5 μL PI and 10 μL Annexin V-FITC, and incubated in the dark at room temperature for 30 min. Cell apoptosis was detected by flow cytometry analysis, and the apoptotic rate was measured using FlowJo version 7.6.1 software (FlowJo, Ashland, OR, USA).

### Western blot

Total protein was extracted from cells lysed with radioimmunoprecipitation buffer (RIPA, Beyotime Biotechnology, Shanghai, China) containing a protease inhibitor cocktail (Beyotime Biotechnology) at 4 °C for 30 min. After centrifugation at 16,000 rpm, 4 °C for 20 min, the protein concentration in the supernatant was measured using the BCA200 protein assay kit (Biosharp Life Science, Hefei, China). An equivalent of 30 μg/lane cellular protein was separated by sodium dodecyl sulfate-polyacrylamide gel electrophoresis (10% SDS-PAGE) and electrotransferred onto a polyvinylidene fluoride membrane (Millipore, USA). After blocking with 5% fat-free milk in Tris-buffered saline/0.05% Tween 20 containing for 1 h, the membranes were probed with primary antibody, according to the manufacturers' recommendations, at 4 °C overnight, followed by incubation with the secondary antibody at room temperature for 1 h. The protein bands were visualized with a chemiluminescence detection kit (Thermo Fisher Scientific Waltham, MA, USA), and images were captured with a scanner using Quality One software (Bio-Rad, Hercules, CA, USA). Image J(NIH, Bethesda, MD) 1.44p software was used for quantitative analysis of the immunoreactive bands.

### Statistical analysis

All analyses were performed using SPSS software version 18.0. Data were presented as mean±standard deviation (SD). All experiments were conducted in triplicate. Group differences were calculated by *t*-test or one-way analysis of variance (ANOVA). Significance was defined as *p*<0.05.

## Results

### PI3K/mTOR and MAPK pathways were activated in SW480 and SW480R cells

**Figure 2:**
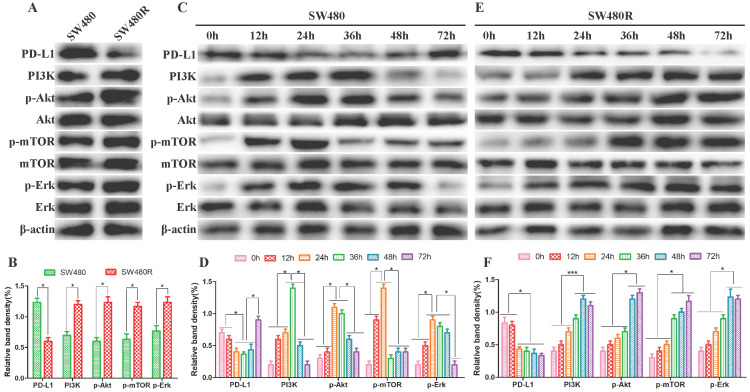
PI3K/mTOR and MAPK pathways were activated in CRC cells. (A and B) The expression of major kinases of the PI3K/mTOR and MAPK pathways in SW480 and SW480R cells was examined by western blot. (C and D) Kinase expression levels in related pathways in SW480 cells. (E and F) Kinase expression levels in related pathways in SW480R cells. Data were expressed as mean±SD of three independent experiments (*p<0.05; **p<0.01; ***p<0.001).

To elucidate the activation status of PI3K/mTOR and MAPK pathways in SW480 and SW480R cells, we analyzed the phosphorylation levels of the main kinases of the related pathways in the two cell lines. Western blot results revealed that the expression of PD-L1 was lower in SW480R than in SW480, indicating that the interaction between PD1 and PD-L1 increased and the function and immune effect of T cells was inhibited. However, the expression of PI3K, p-Akt, p-mTOR, and p-ERK was higher in SW480R cells than in SW480 cells (**Figure 2A, Figure 2B**), indicating that the PI3K/mTOR and MAPK pathways were activated through PD1/PD-L1. Next, SW480 and W480R cells were treated with BMS-1166 for 0, 12, 24, 36, 48, and 72 h to assess the activated form of the two pathways. Western blot results showed that the phosphorylation levels of major kinases in the PI3K/mTOR and MAPK pathways were progressively elevated in BMS-1166-treated SW480 cells. However, after reaching the peak at 24 h, the expression of these molecules declined to control levels (*p*<0.05), but that of PD-L1 recovered to the control level after a transient decrease. These findings suggested that BMS-1166 induces stress activation of the PI3K/mTOR and MAPK pathway in SW480 cells (**Figure 2C, Figure 2D**). In addition, the effect of BMS-1166 on SW480R cells stably activated the key molecules in PI3K/mTOR and MAPK pathways after 24 h and also activated the expression of PD-L1 (**Figure 2E, Figure 2F**). Thus, we speculated that the BMS-1166 resistance to CRC may be attributed to the stable activation of PI3K/mTOR and MAPK signaling pathways.

### BEZ235 inhibits the activation of PI3K/mTOR and MAPK pathway in CRC cells

**Figure 3:**
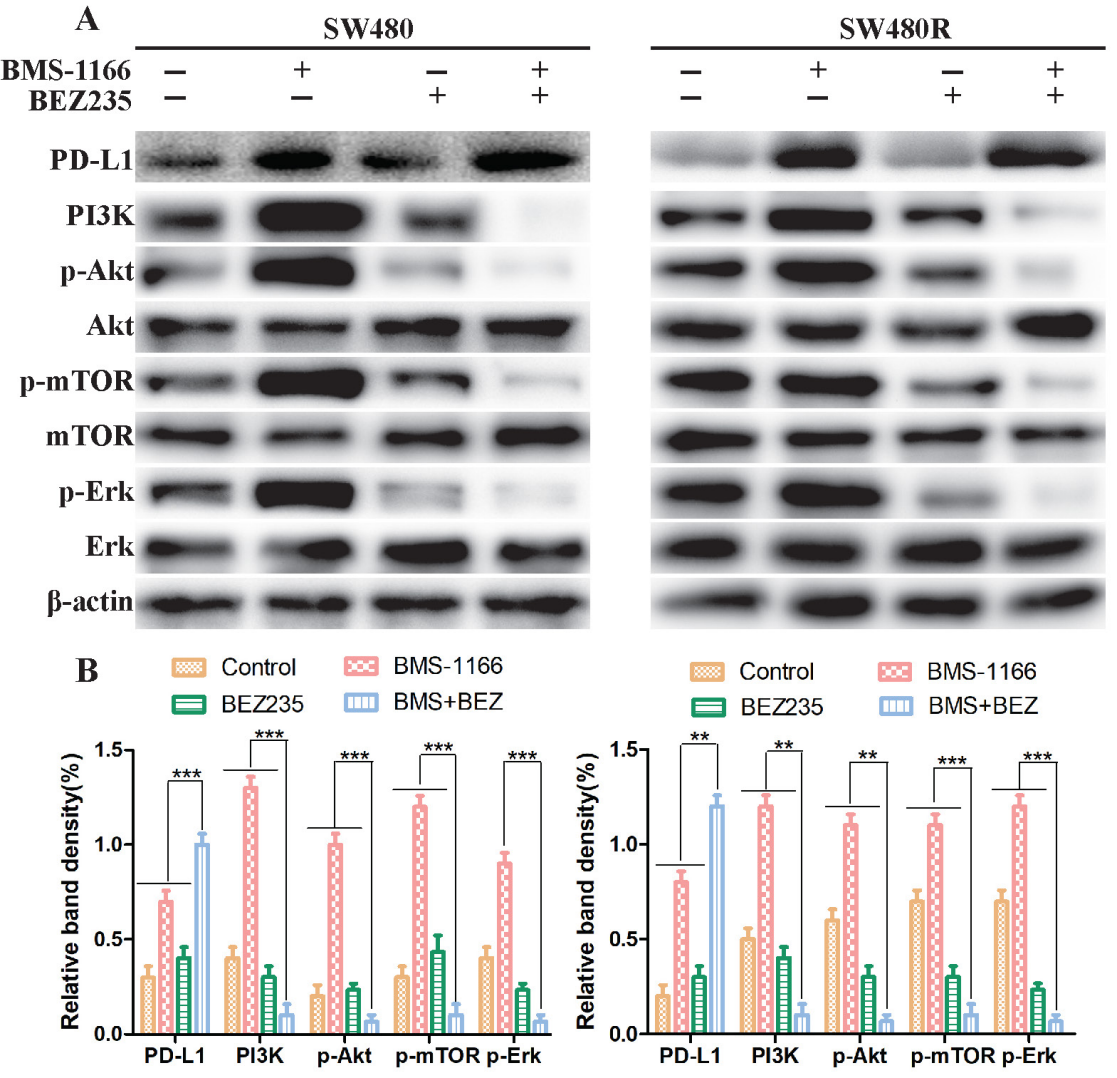
BEZ235 increases the treatment effect by blocking the PI3K/mTOR and MAPK pathways in CRC cells. (A and B) The protein analysis of PI3K/mTOR and MAPK pathways in SW480 and SW480R cells treated with BMS-1166 (SW480: 0.5 μM; SW480R: 1 μM), BEZ235 (0.5 μM), or the combination of two drugs for 24 h. Data were expressed as mean±SD of three independent experiments (*p<0.05; **p<0.01; ***p<0.001).

Next, we explored the mechanism of BEZ235 combined with BMS-1166 in SW480 and SW480R cells. To determine whether the effects of combined BMS-1166 and BEZ235 block the PI3K/mTOR and MAPK pathway activation in CRC cells by suppressing PD1 binding to PD-L1, we detected the levels of PD-L1 and key molecules of PI3K/mTOR and MAPK signaling after 24-h drug treatment by western blot. The results showed that BMS-1166 promoted PI3K, Akt, mTOR, and Erk phosphorylation. BEZ235, in combination with BMS-1166, reduced the expression of the PI3K, p-Akt, p-mTOR, and p-Erk in SW480 and SW480R cells compared to BMS-1166 or BEZ235 single treatment by inhibiting the binding of PD1 to PD-L1 (**Figure 3A, Figure 3B**). Thus, it can be deduced that BEZ235 enhances the effect of BMS-1166 treatment by blocking the PI3K/mTOR pathway and interfering with the crosstalk of the MAPK pathway.

### BEZ235 combined with BMS-1166 inhibits CRC cell proliferation

**Figure 4:**
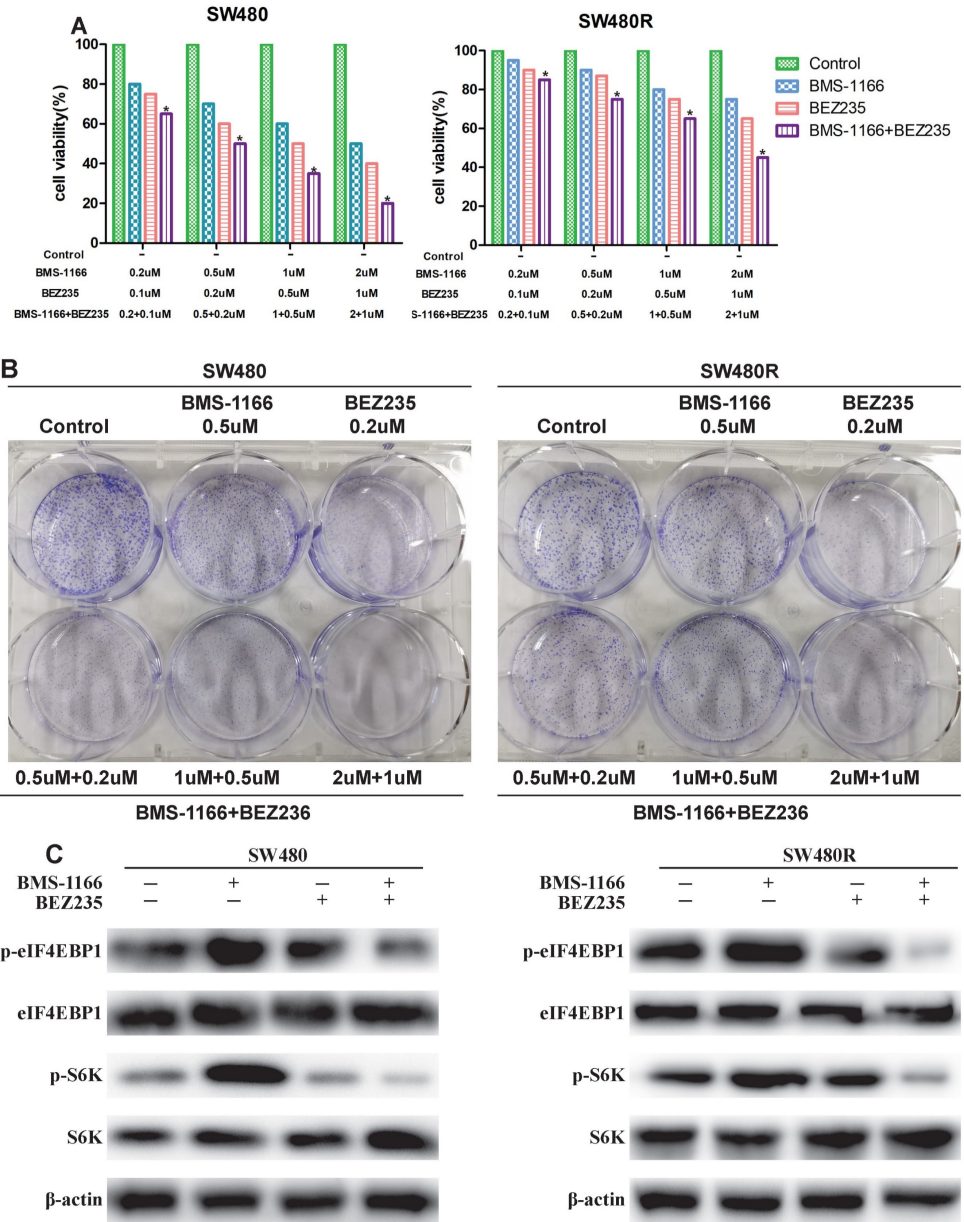
BEZ235 enhances the anti-proliferative effects of BMS-1166 in CRC cells. (A) Cell viability of SW480 and SW480R cells after exposure to BMS-1166 for 24 h. (B) Cell viability of SW480 and SW480R cells treated with each group after 24 h was assessed by MTT assays. (C) Colony formation assays were used to determine the clonogenic capacity of SW480 and SW480R cells treated with each group. (D) Expression and phosphorylation of S6K and elF4EBP1 after treatment with BEZ235 plus BMS-1166 in SW480 and SW480R cells. Data were expressed as mean±SD of three independent experiments (*p<0.05; **p<0.01; ***p<0.001).

To detect the inhibitory effects on CRC cells treated with BMS-1166, BEZ235, or a combination of the two drugs, we measured the cell viability by MTT assay. BMS-resistant cell SW480R sub-cell lines were established by exposure to escalating doses of BMS-1166, and the IC50 values were determined (**Figure 4A**). The viability of SW480 and SW480R cells treated with BMS-1166, BEZ235, and the combination of the two drugs decreased in a dose-dependent manner (**Figure 4B**). Compared to BMS-1166 or BEZ235 monotherapy, combination therapy has a more significant inhibitory effect on cell viability. The results of colony formation assays also showed that BMS-1166 plus BEZ235 had a higher inhibitory effect on clone formation than the control group and any single-agent treatment (**Figure 4C**). Furthermore, we assessed whether BEZ235 inhibits CRC cell proliferation by modulating the PI3K/mTOR pathway. The results showed that BEZ235 combined with BMS-1166 inhibits the phosphorylation of the downstream effector molecules (S6K and elF4EBP1) of the PI3K/mTOR pathway in CRC cell lines (**Figure 4D**). These results confirmed that BEZ235 plus BMS-1166 suppresses cell viability by blocking the PI3K/mTOR pathway in SW480 and SW480R cells.

### BEZ235 combined with BMS-1166 inhibits CRC cell migration

**Figure 5:**
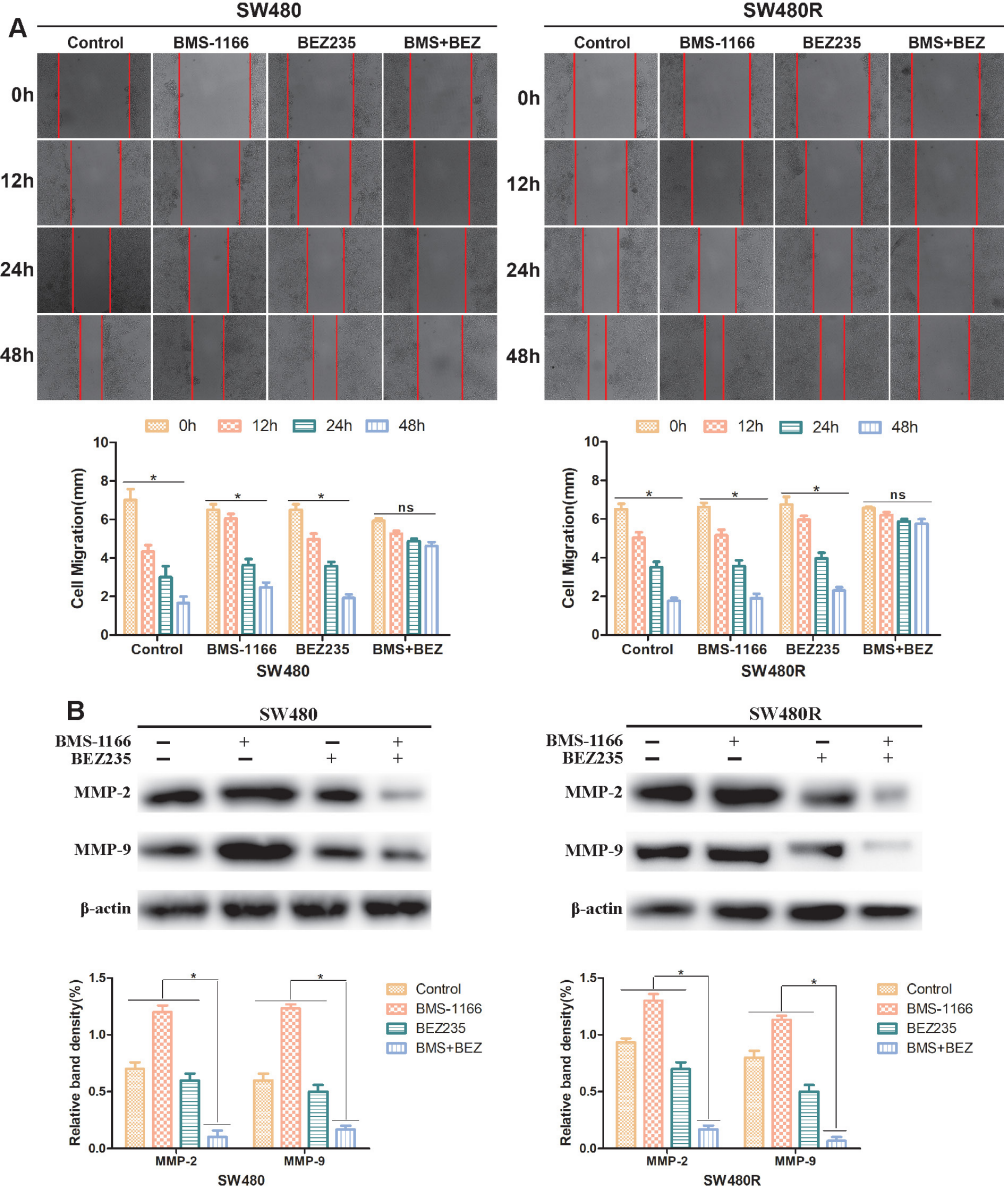
BEZ235 increases BMS-1166 inhibition of SW480 and SW480R cell migration. (A) Wound healing assays were carried out to assess the migration ability of CRC cells treated with BMS-1166, BEZ235, or the combination of the two drugs for 12, 24, and 48 h. (B) Proteins related to tumor cell migration (MMP-2 and MMP-9) were detected by western blot. Data were expressed as mean±SD of three independent experiments (*p<0.05; **p<0.01; ***p<0.001).

To explore the effect of BEZ235 plus BMS-1166 on CRC cell migration ability, SW480 and SW480R cells were treated with BMS-1166, BEZ235, or a combination of the two for 0, 12, 24, and 48 h by wound healing test. The migration inhibition rates of BEZ235 combined with BMS-1166 were significantly higher than those of either single agent group (**Figure 5A**). Western blot results revealed that after treatment with BMS-1166 combined with BEZ235, the expressions of MMP-2 and MMP-9 in SW480 and SW480R cells were significantly lower than with monotherapy treatment (**Figure 5B**). These findings indicated that BEZ235 combined with BMS-1166 suppresses SW480 and SW480R cell migration.

### Pro-apoptotic effect of BEZ235 combination of BMS-1166 on CRC cells

**Figure 6:**
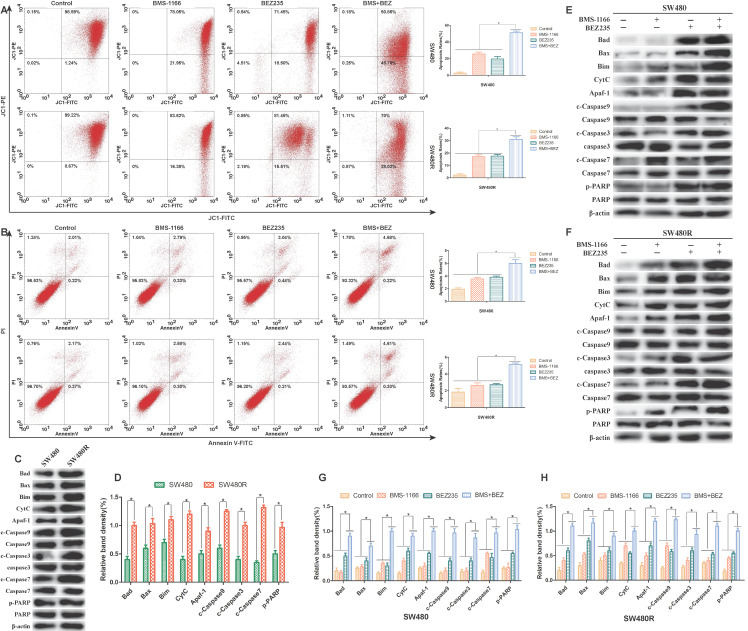
Flow cytometry and protein analysis of the pro-apoptotic effect of BMS-1166 and BEZ235 in SW480 and SW480R cells. (A) CRC cells were treated with each treatment group for 24 h, stained with JC-1, and detected by flow cytometry. (B) CRC cells were treated with each treatment group for 24 h, stained with Annexin V-FITC/PI, and detected by flow cytometry. (C and D) Western blot analyzed the phosphorylation levels of the main pro-apoptosis protein in the two cell lines, (E-H) Total protein extract from each group of drug-treated SW480 and SW480R cells. The expression levels of mitochondria-associated pro-apoptotic proteins (Bad, Bax, Bim, CytC, and Apaf-1) and caspase proteins (caspase-9, -3, -7, and PARP) in each treatment group were analyzed by western blot. Data were expressed as mean±SD of three independent experiments (*p<0.05; **p<0.01; ***p<0.001).

To assess the ability of BEZ235 plus BMS-1166 to induce CRC cell apoptosis, the pro-apoptotic effects of the various treatments were examined by flow cytometry(JC-1, AnnexinV-FITC/PI staining) and western blot. 24 h post-drug treatment, JC-1 flow cytometry results showed that BMS-1166 and BEZ235 induced apoptosis independently in SW480 and SW480R cells, and the combination of two drugs had an enhanced pro-apoptotic effect (**Figure 6A**). AnnexinV-FITC/PI flow cytometry showed similar pro-apoptotic effects (**Figure 6B**). To learn the expression of pro-apoptosis-related proteins in SW480 and SW480R cells, we analyzed the phosphorylation levels of the main pro-apoptosis in the two cell lines. Western blot results revealed that the expression of Bad, Bax, Bim, CytC, Apaf-1, caspase-9, caspase-7, and caspase-3 was higher in SW480R cells than in SW480 cells (**Figure 6C, Figure 6D**). The overexpression of Bad, a key regulatory protein in the mitochondrial apoptotic pathway, leads to apoptosis by suppressing the activity of the anti-apoptotic protein Bcl-xL and further promotes the release of cytochrome C and cascade activation of caspase-9, caspase-3, and caspase-7. Western blot results revealed that the expressions of pro-apoptosis-related proteins, Bad, Bax, Bim, CytC, Apaf-1, caspase-9, caspase-3, caspase-7, and PARP were increased in SW480 and SW480R cells in response to the combination of the two drugs (**Figure 6E-H**). These findings indicated that the pro-apoptotic effect of BEZ235 combined with BMS-1166 is superior to a single treatment with these drugs in CRC cells.

## Discussion

CRC is driven by genetic and epigenetic changes in colonic epithelial cells and tumor-host interaction^19^. With the occurrence and development of tumors, the combination of PD-1 and PD-L1 inhibit the immunity of cells in the host, which leads to the immune escape of tumor cells^20^,^21^. Tumor-associated macrophages (TAMs) participate in M1-like macrophages that promote anti-tumor immunity and M2-like macrophages with cancer-promoting properties. M2-like macrophages mediate the immune escape of tumor cells through PD-1 and are activated by interleukin (IL)-4, IL-10, IL-13, or colony-stimulating factor 1 (CSF-). M2-like macrophages participate in wound healing and tissue repair and mediate anti-inflammatory responses by producing anti-inflammatory cytokines, including IL-10. Due to the influence of tumor microenvironment (TME), the M1 and M2 TAMs in tumors are conducive to the proliferation of tumor cells. This heterogeneity limits the clinical use of PD-1/PD-L1 inhibitors alone^22^. A comprehensive search of PD‑1/PD‑L1 inhibitor monotherapy or combination therapy in neoadjuvant settings of 11 types of solid cancers was performed using the PubMed database. The data presented in 99 clinical trials indicated that PD‑1/PD‑L1 combined therapy, especially immunotherapy plus chemotherapy, improved postoperative disease‑free survival compared to those without pathological remission. Therefore, blocking the binding of PD-1 and PD-L1 restores T cell function, enhances tumor immune response, and inhibits tumor cell growth, thus suppressing metastasis.

One of the mechanisms for tumor cell-stimulated expression of PD-L1 is the activation of EGFR, PI3K/mTOR, or MAPK signaling pathways^23^. In the current studies, BEZ235 combined with BMS-1166 reduced PD-L1 expression and inhibited PI3K/mTOR and MAPK activation. Thus, PI3K/mTOR and MAPK appear to play an essential role in the inhibitory effect on BMS-1166-blocked PD-L1 expression in CRC cells.

Furthermore, the PI3K/mTOR pathway plays a key role in cell proliferation, survival, metabolism, and migration and is one of the most commonly dysregulated oncogenic pathways in cancers, including CRC. Genetic alterations, such as activating mutations, amplification of PIK3CA and AKT1, and the loss-of-function mutations of PTEN, served as potential predictive biomarkers for treatment selection^24^. BEZ235, a dual ATP-competitive PI3K and mTOR inhibitor, blocks PI3K and mTOR kinase activity, primarily through ATP binding these enzymes. The combination of BEZ235 with BMS-1166 reduces the phosphorylation level of mTOR downstream S6K and eIF4EBP1, thus inhibiting the proliferative viability and clonogenic ability of CRC cells^25^.

Some studies have shown that MMP2 and MMP9 participate in angiogenesis by destroying basal-layer molecules and remodeling the extracellular matrix during angiogenesis^26^. The MMPs in the extracellular matrix acquire the migration characteristics facilitating tumor cell invasion into the surrounding tissues and metastasis to the secondary sites^27^. Our results demonstrated that when CRC cells were exposed to the combination of BEZ235 and BMS-1166, the wound size in the wound-healing assays was significantly more than with BMS-1166 or BEZ235 monotherapy, and the expression levels of MMP-2 and MMP-9 were reduced.

PI3K/Akt signaling is closely related to the mitochondrial apoptosis pathway. p-Akt phosphorylated Bad, a member of the Bcl-2 family, followed by Bcl-xL-induced anti-apoptosis^28^. We also observed that BEZ235 plus BMS-1166 treatment inhibited the expression of p-Akt and p-Erk in CRC cells but upregulated the expression of mitochondrial apoptotic proteins, such as Bad, Bax, and Bim. Concurrently, the mitochondrial membrane potential was depolarized, and CytC was released into the cytoplasm from the mitochondria, whereby it formed an apoptotic complex with caspase-9 through multimerization of Apaf-1 factor. Caspase-9 cleaves and activates the downstream actions of caspase-3 and -7 to promote apoptosis. Thus, BEZ235 plus BMS-1166 inhibited the PI3K/mTOR and MAPK pathways, triggering the mitochondrial apoptotic pathway and thus enhancing the anti-tumor effect of BMS-1166.

## Conclusions

The current study indicated that by inhibiting the combination of PD1 and PD-L1, T cell function was restored, abnormal activation of PI3K/mTOR and MAPK pathways was suppressed, CRC cell proliferation and migration was reduced, and apoptosis was promoted. These findings would provide a theoretical basis for BEZ235 plus BMS-1166 in CRC drug resistance.

## Abbreviations

CRC: Colorectal Cancer
PD-L1: programmed cell death ligand1
PD-1: programmed cell death receptor1
PBS: phosphate buffered saline
FBS: Fetal Bovine Serum
JC-1: 5,5',6,6'-tetrachloro-1,1',3,3'-tetrae-thyl-benzimidazolyl carbocyanine iodide
FITC: fluorescein isothiocyanate
MTT: 3-(4,5-dimethyl-2-thiazolyl)-2,5-diphenyl-2-H-tetrazolium bromide
SDS-PAGE: sodium dodecyl sulfate-polyacrylamide gel electrophoresis
